# Missed opportunities for early infant diagnosis of HIV in rural North-Central Nigeria: A cascade analysis from the INSPIRE MoMent study

**DOI:** 10.1371/journal.pone.0220616

**Published:** 2019-07-31

**Authors:** Udochisom C. Anaba, Nadia A. Sam-Agudu, Habib O. Ramadhani, Nguavese Torbunde, Alash’le Abimiku, Patrick Dakum, Sani H. Aliyu, Manhattan Charurat

**Affiliations:** 1 International Research Center of Excellence, Institute of Human Virology Nigeria, Abuja, Federal Capital Territory, Nigeria; 2 Division of Epidemiology and Prevention, Institute of Human Virology, University of Maryland School of Medicine, Baltimore, Maryland, United States of America; 3 Prevention, Care and Treatment Unit, Institute of Human Virology Nigeria, Abuja, Federal Capital Territory, Nigeria; 4 Office of the Director-General, National Agency for the Control of AIDS, Abuja, Nigeria; The Ohio State University, UNITED STATES

## Abstract

**Background:**

Early identification of HIV-infected infants for treatment is critical for survival. Efficient uptake of early infant diagnosis (EID) requires timely presentation of HIV-exposed infants, same-day sample collection, and prompt release of results. The MoMent (Mother Mentor) Nigeria study investigated the impact of structured peer support on EID presentation and maternal retention. This cascade analysis highlights missed opportunities for EID and infant treatment initiation during the study.

**Methods:**

HIV-infected pregnant women and their infants were recruited at 20 rural Primary Healthcare Centers. Routine infant HIV DNA PCR testing was performed at centralized laboratories using dried blood spot (DBS) samples ideally collected by age two months. EID outcomes data were abstracted from study case report forms and facility registers. Descriptive statistics summarized gaps and missed opportunities in the EID cascade.

**Results:**

Out of 497 women enrolled, delivery data was available for 445 (90.8%), to whom 415 of 455 (91.2%) infants were live-born. Out of 408 live-born infants with available data, 341 (83.6%) presented for DBS sampling at least once. Only 75.4% (257/341) were sampled, with 81.7% (210/257) sampled at first presentation. Only 199/257 (77.4%) sampled infants had results available up to 28 months post-collection. Two (1.0%) of the 199 infants tested HIV-positive; one infant died before treatment initiation and the other was lost to follow-up.

**Conclusions:**

While nearly 85% of infants presented for sampling, there were multiple missed opportunities, largely due to health system and not necessarily patient-level failures. These included infants presenting without being sampled, presenting multiple times before samples were collected, and getting sampled but results not forthcoming. Finally, neither of the two HIV-positive infants were linked to treatment within the follow-up period, which may have led to the death of one. To facilitate patient compliance and HIV-free infant survival, quality improvement approaches should be optimized for EID commodity availability, consistent DBS sample collection, efficient processing/result release, and prompt infant treatment initiation.

## Introduction

The persistence of HIV transmission between women living with HIV and their HIV-exposed infants (HEI) is a serious public health concern. If left untreated, half of all HIV-infected infants will die before reaching two years of age [1]. Reduction of infant mortality is a key focus of the United Nation’s Sustainable Development Goals (SGDs) [2]. In high HIV–burden countries, vertically-transmitted HIV contributes significantly to infant mortality rates [3]. Prevention of mother-to-child-transmission of HIV (PMTCT) programs aim to reduce and ultimately eliminate vertical transmission of HIV, and to facilitate maternal and infant health and survival [4, 5]. Thus, the PMTCT agenda aligns with the SDG agenda in their mutual objective of reducing maternal and infant mortality globally.

PMTCT strategies are targeted to four main mother-infant life-stages: the preconception, antenatal, labor/delivery and postnatal periods [6, 7]. These 4 stages span a comprehensive PMTCT cascade of care for the HIV-infected mother and exposed infant [7, 8]. Postnatal PMTCT strategies include early identification of HIV-infected children to ensure survival and maximize quality of life [6, 7]. Early infant diagnosis (EID) is recommended by the World Health Organization (WHO) and is defined as the receipt of a virological HIV test by 2 months of age for all HEI [5]. The Global Plan towards the elimination of new HIV infections among children by 2015 and keeping their mothers alive targeted improvements in PMTCT program performance including EID uptake in 21 PMTCT priority countries [4]. In the Global Plan’s final report, estimated EID uptake among these countries ranged between 2% to >95%, with Burundi (2%), Chad (3%) and Nigeria (9%) in the bottom 3 for this indicator [4]. By the end of 2017, estimated EID uptake for Nigeria was only slightly improved at 12% [9].

In spite of a relatively low 2017 HIV prevalence of 2.8% [10], Nigeria’ population of 196 million [11] contributes to its large HIV and PMTCT burden. More than 6 million Nigerian women are pregnant every year, and nearly 200,000 of them are HIV-infected women [12]. These women, along with their infants, present a large target population for PMTCT interventions in Nigeria. Antiretroviral drugs (ARVs) play a critical role in reducing the risk of mother-to-child transmission of HIV (MTCT) [13–15]. Yet, only 32% of pregnant women living with HIV in Nigeria received ARVs for PMTCT in 2016; the lowest amongst all Global Plan countries [4, 16]. Furthermore, Nigeria had the highest six week (13.1%) and final MTCT rates (23.0%) among Global Plan countries [4].

In line with WHO recommendations, Nigeria’s HIV guidelines have also recommended EID by two months of age for HEI, with sampling targeted between six and eight weeks of age [17–19]. Furthermore, the 2010–2015 and 2017–2021 National Strategic Frameworks for PMTCT aimed to increase infant access to early diagnostic services and ARV prophylaxis to 80% by 2015 and 95% by 2021 [17, 20]. Unfortunately, uptake of EID and ARV prophylaxis among HEI and rates of antiretroviral therapy (ART) initiation amongst HIV-infected infants continues to be sub-optimal. By the end of 2017, in addition to the low proportion of HEI receiving EID, only 26% of eligible HIV-infected Nigerian children received ART [21].

The extent and persistence of these PMTCT program gaps warrant a detailed evaluation of bottlenecks that serve as barriers to accelerated progress. This is important not only to Nigeria, but to the entire West and Central African region, which lags behind the rest of sub-Saharan Africa in HIV outcomes and targets, including PMTCT [22, 23]. The INSPIRE MoMent implementation research study evaluated the impact of structured peer support on presentation for EID and maternal retention in care among mother-infant pairs in rural Nigeria [24–26]. INSPIRE (Integrating and Scaling up PMTCT through Implementation Research) was an innovative five-year (2012–2017) Global Affairs Canada and WHO initiative for implementation research in three high PMTCT-burden African countries: Malawi, Nigeria and Zimbabwe [27–29]. MoMent Nigeria was one of six INSPIRE awards and one of the two implemented in Nigeria. As MoMent data were collected to report study outcomes, data on programmatic events and procedures were also collected to contextualize the study environment. This paper examines the programmatic aspect of the EID presentation outcome and provides a cascade analysis of EID presentation, testing and result delivery for all infants enrolled in the study.

## Materials and methods

### Study setting and population

The MoMent Nigeria prospective cohort study was conducted at 20 Primary Health Care centers (PHCs) in the Federal Capital Territory (FCT) and Nasarawa states in North-Central Nigeria. In 2014, HIV sero-prevalence among pregnant women in FCT and Nasarawa were 5.8% and 6.3% respectively, against a national figure of 3.0% [30], showing relatively high sub-national HIV burden in these states. PMTCT and EID services at all study sites were supported by the United States’ President’s Emergency Plan for AIDS Relief (PEPFAR) through the Institute of Human Virology Nigeria (IHVN), a local non-governmental organization [31]. The PHCs were located in 20 different rural and semi-rural towns and villages across 9 local government areas (districts) in the two study states. The distance between study PHCs and Nigeria’s capital city Abuja in FCT ranged from ~15 km to 230 km.

The study enrolled and followed-up mother-infant pairs between April 2014 and November 2017. Criteria for site selection and participant recruitment have been detailed in previous publications [24, 32]. Briefly, HIV-infected pregnant women aged 15 years and older were consecutively recruited during presentation for antenatal care (ANC) at pair-matched study PHCs. Eligibility criteria included agreeing to being assigned HIV-infected counselors, known as Mentor Mothers in the intervention arm, and a routine peer supporter in the control arm. Mentor Mothers provided peer support via a structured program with baseline training and a detailed scope of work, close supervision of Mentor Mother field activities, a standardized logbook for recording client interactions, a missed appointment tracking procedure, and periodic performance evaluations [24]. In the control arm, routine peer support was provided also by HIV-infected peer counselors through the existing program, however there was little training, structure or supervision.

As part of routine PMTCT service appointments, it is recommended that HIV-exposed infants present for EID by the WHO-recommended age of two months [18, 19, 33]. Nigerian guidelines recommend for this to occur at six weeks of life for the exposed infant, which conveniently coincides with the initiation of cotrimoxazole prophylaxis as well as receipt of infant immunizations [18, 19].

### Ethical approvals

The study was approved by the Nigerian National Health Research Ethics Committee, the Ethics Review Committee of the World Health Organization, and the Institutional Review Board of the University of Maryland Baltimore. Our target population was pregnant women 15 years and older, and minors under 18 years were allowed by all three ethics committees to provide consent for themselves on account of pregnancy and/or motherhood. Written informed consent was therefore obtained from all enrolled pregnant women, who also provided written consent for enrollment of their live-born infants.

### Sample collection and processing

Upon presentation for the DNA PCR sample collection, whole blood samples were collected from a heel or toe prick of each infant and spotted onto five circles on filter paper for overnight drying at ambient temperature. Labeled dried blood spot (DBS) filter paper samples were sealed in humidity-free bags and transported to a PCR laboratory for processing. Based on a geographical hub-and-spoke model, two laboratories were programmatically responsible for DNA PCR processing for the 20 study sites and both were located in the Federal Capital Territory (FCT); one in central Abuja and the other in Gwagwalada, approximately 60 km west of Abuja.

Out of 20 study sites, 11 were in FCT, and nine in Nasarawa. There were no DNA PCR processing laboratories in Nasarawa state during the study, therefore all samples from Nasarawa study sites were routinely transported to the central Abuja laboratory for processing. DBS samples were routinely collected by IHVN PMTCT staff and transported to processing labs via either official or commercial vehicle.

Upon receipt by the processing laboratory, one spot from each filter paper was tested by COBAS Ampliprep/Taqman HIV-1, version 2.0 real-time PCR assay (Roche Diagnostics, Indianapolis, USA), according to manufacturer instructions. For confirmation, all samples testing positive were retested using a second DBS (taken from the same filter paper sample) prior to issuing final results. HIV-negative DNA PCR samples were not retested.

### Data collection and analysis

Data on DBS sample collection, transport, processing, results notification logistics and procedures were obtained from IHVN Standard Operating Procedures. Outcome data for the MoMent study’s EID cascade analysis were abstracted from study case report forms and DNA PCR result registers located in the processing laboratories as well as at the study sites. For the MoMent study’s outcome analysis, successful EID presentation was defined as presentation for DNA PCR sample collection between 35 and 62 days (5 and 8 weeks) of age [34]. This aligns with the previously-mentioned national recommendation for EID presentation at six weeks of life for convenience. Additionally, MoMent’s “EID presentation” definition conforms to WHO recommendations for infants to present earliest at 4 weeks of age and latest by age two months [33]. HEI presentation for sample collection after the EID period was described simply as “DNA PCR presentation”. Uptake was defined as the successful collection of a DNA PCR blood sample from the presenting HEI. Reasons for infant presentation to facility were obtained from accompanying caregivers and documented in facility registers and study forms for each visit. For an infant making multiple visits, only visit data for which DBS was the reason or where it was collected, was included for analysis.

To evaluate for gaps and missed opportunities in MoMent’s EID cascade, HEI presentation for, and uptake of HIV testing was analyzed in four domains:

- Proportion of live-born HEI presenting for first DNA PCR regardless of age/timing;
- Proportion of infants who ultimately had the first DBS sample collected regardless of the number of times they presented before successful collection;
- Proportion of presenting HEI with DBS samples collected at first presentation;
- Proportion of all HEI with samples collected who had results available at the testing laboratories at least three months post sample collection.

Additionally, data from the above four domains were secondarily analyzed by study arm using Chi-square, to determine the impact, if any, of the structured Mentor Mother intervention on the EID cascade beyond presentation by two months of age. P values less than 0.05 were considered statistically significant. Analysis was performed using STATA version 15 (StataCorp LP, College Station, TX). Only data for the first DNA PCR tests successfully collected were analyzed. All infants were followed up for a minimum of 12 months post-delivery.

## Results

A total of 497 HIV-infected pregnant women were enrolled from the 20 study PHCs. Baseline characteristics of enrolled women and live-born infants have been reported previously [32]. Briefly, out of 445 women’s deliveries for which data were available, 415 of 455 (91.2%) infants (including 10 sets of twins) were live-born [32, 34]. Data from seven infants were not available, thus analysis was performed for 408 live-born infants.

### First DNA PCR presentation among infants

Out of 408 live-born infants with data available, 341 (83.6%) presented for DBS sample collection at least once, irrespective of timing (Table 1). Factors associated with timely presentation for EID in the MoMent study have been reported elsewhere [34]. The youngest presenting HEI was nine days old, however the youngest infant to have a sample collected for DNA PCR was 12 days old. The oldest HEI to present for first DBS sample collection was 595 days (19.8 months) old, which defined the end of the “first DNA PCR” assessment period for the study. Nearly three-fourths (246/341, 72.2%) of infants presented during the appropriate EID period, per study definition. A total of 35/408 (8.6%) infants had died by the end of the DNA PCR assessment period, including 14 (4.1%) of the 341 presenting infants (Table 1).

**Table 1:** First presentation for DNA PCR sample collection among live-born HIV-exposed infants, N = 341.

Post-delivery period	No. of infants presenting n (%)^a^	Age at first presentation, median (IQR)	No. of infants with DBS sample collected at first presentation n (%)^b^	No. of deaths among presenting infants^c^	No. of non-presenting infants who died^d^
0–34 days	9 (2.6)^e^	28 days (16–32)	4 (44.4)^e^	0	17
35–62 days	246 (72.2)	44 days (42–48)	142 (57.7)	2	1
63–595 days^f^	86 (25.2)	139 days (77–192)	64 (74.4)	12	3
**Total**	**341 (100.0)**	**46 days (42–62)**	210 (61.6)	**14**	**21**

IQR: interquartile range

^a^Presenting for DNA PCR for the first time

^b^Row percentage; denominator is number of infants presenting

^c^Presented and died later in study follow-up period. Post-delivery period categorization relevant to timing of death

^d^Never presented; died in study follow-up period. Post-delivery period categorization relevant to timing of death

^e^Three of remaining 5 infants who presented early with no sample collected returned later in eligible time-period; all 3 had samples collected. The last 2 infants never returned for DNA PCR.

^f^595 days is age of oldest child to present for HIV testing for the first time

### The MoMent study’s EID cascade

**Fig 1:**
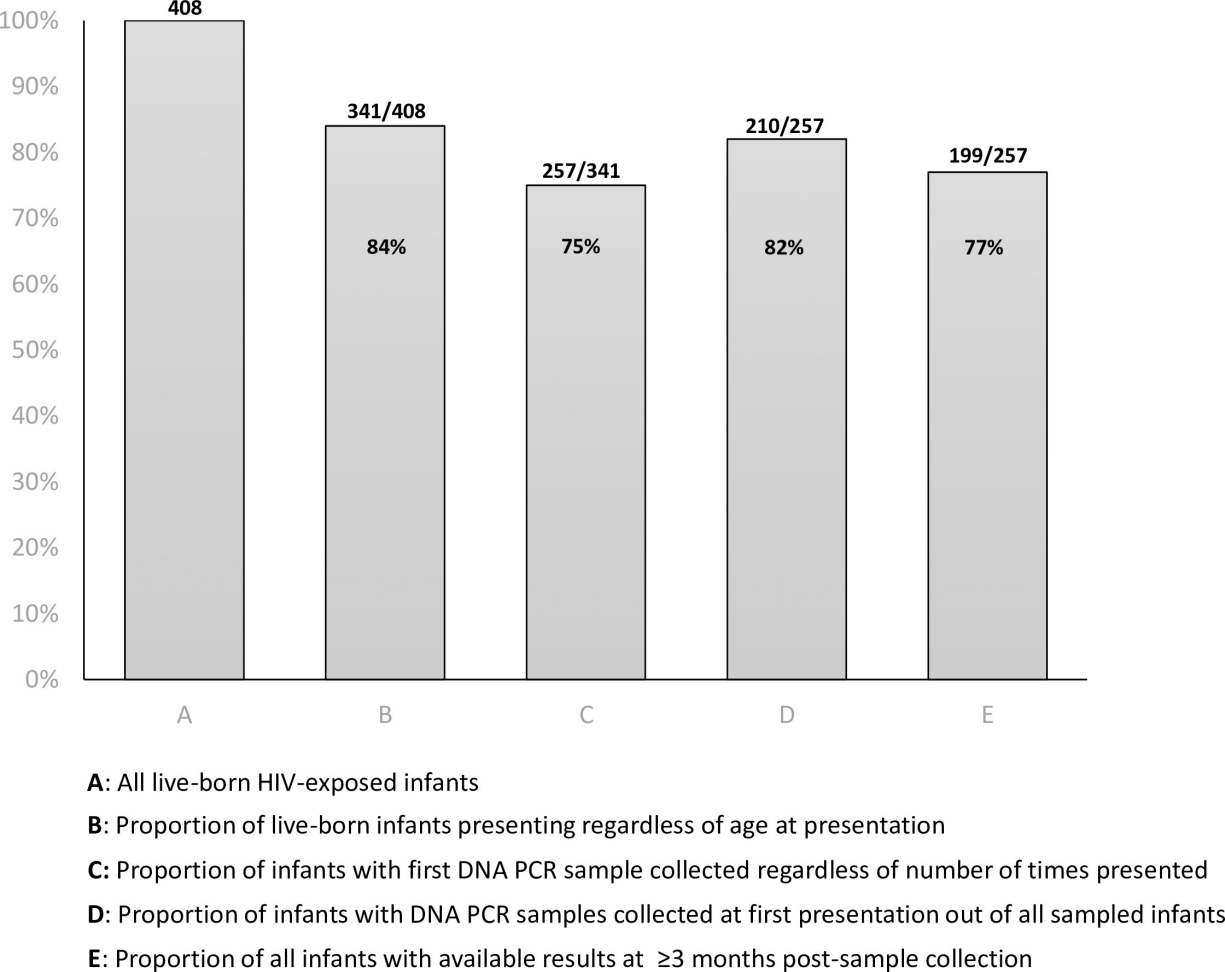
Early infant diagnosis cascade for MoMent HIV-exposed infants, N = 408.

Out of the 341 HEI who presented, 257 (75.4%) had first DBS samples collected regardless of number of presentations (Fig 1). This indicates that 84 (24.6%) infants who presented never had their samples collected at their study site during the assessment period.

Amongst the 257 presenting infants who were successfully sampled, 210 (81.7%) had samples collected at first presentation whereas 47 (18.3%) had their samples collected on a subsequent presentation. From the 257 DNA PCR samples collected for processing, only 199 (77.4%) results were available from the laboratories (not the facilities) at a minimum of three months and up to 28 months post-collection, during the follow-up period (Fig 1). Furthermore, 2 (1.0%) of the 199 HEI who had available results tested HIV-positive. Lastly, out of the 199 results available, 127 (63.8%) were sampled within the study’s “early presentation” period of 35–62 days of age.

**Fig 2:**
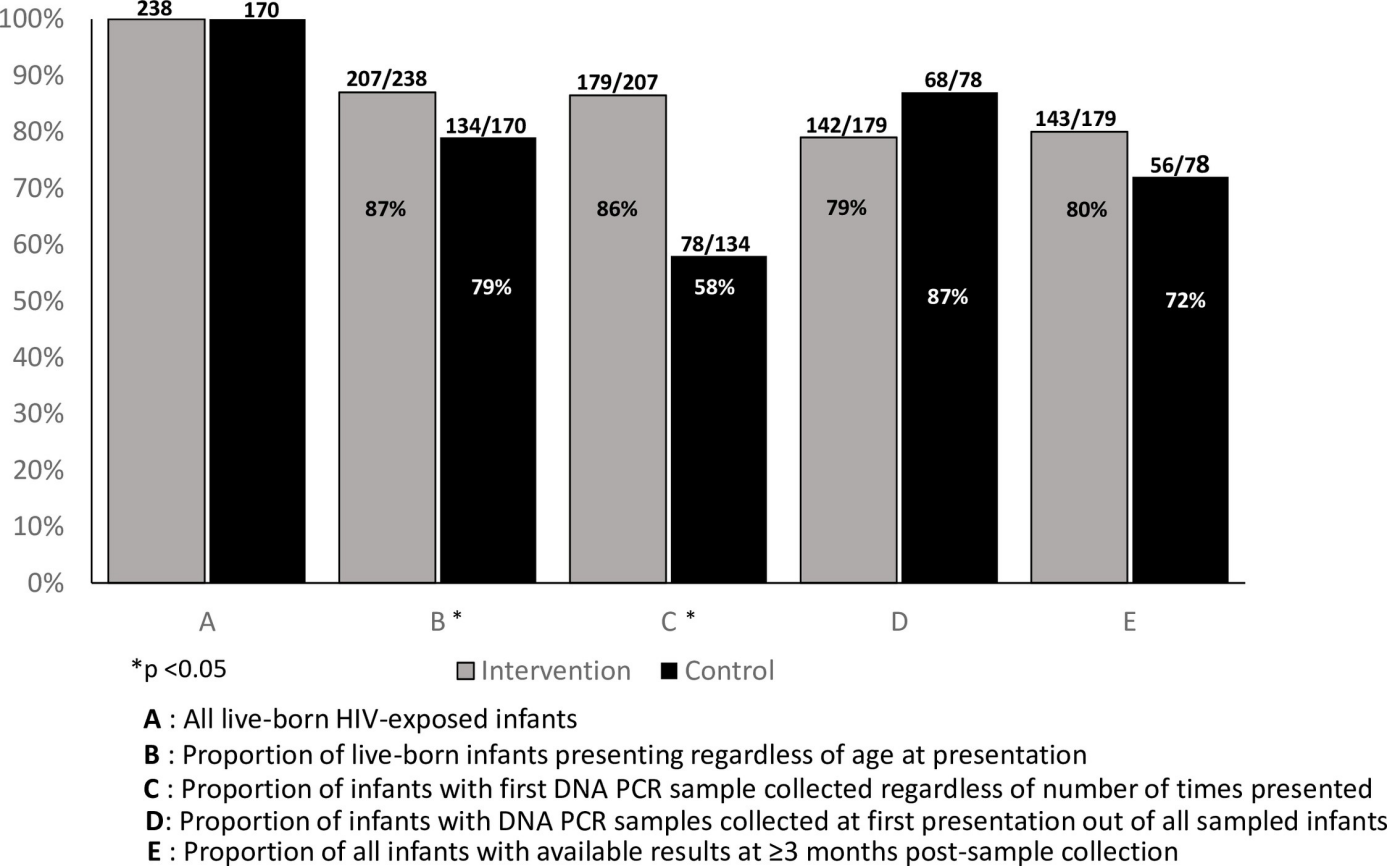
Early infant diagnosis cascade for MoMent HIV-exposed infants by study arm.

A secondary analysis was performed to compare EID cascade outcomes by study arm (Fig 2).

Fig 2 shows that a significantly higher proportion of infants exposed to the structured MM intervention (87%) presented for DNA PCR sample collection compared to control (79%), regardless of age at presentation (p = 0.03). Similarly, regardless of the number of times presented, more infants presenting from the intervention arm (86%) had their samples collected compared to those in the control arm (58%, p<0.001). However, there were no significant differences in terms of proportion of infants with samples collected at first presentation (p = 0.1) and with results available at the testing laboratory at least 3 months post-sample collection (p = 0.2).

### Outcomes for infants testing HIV-positive

Outcomes for the two infants testing HIV-positive are displayed in Table 2. One infant died and there was no information available for the second infant beyond presentation for DNA PCR around six weeks of life.

**Table 2:** Follow-up and linkage to care for infants testing positive at first DNA PCR, N = 2.

	Study ID/Arm^a^	Age at Sample Collection	Age Result Available at Study PHC	Linkage and Follow-up Data
**Infant 1**	KOB14B	6wks 4 days	Result received but date not documented	MIP reported as LTFU; no further information available
**Infant 2**	KTK09B	6wks 6 days	Staff report that PHC did not receive result	MIP LTFU initially, returned to PHC when infant 18 months old and ill. Rapid HIV test positive. Immediate referral to tertiary facility but infant died same day.

^a^ Study arm: Control = A; Intervention = B

PHC: primary healthcare center; MIP: Mother-infant pair, LTFU: lost to follow-up

### Fetal and infant death

There were 455 fetuses/infants from 445 pregnant women with available delivery information. Table 3 displays the timing of fetal and infant death for this cohort, reported as miscarriage (spontaneous abortion), stillbirth, neonatal and post-neonatal losses according to WHO definitions [35]. Specific causes of death were not available due to limited investigation/documentation of fetal and infant death at study facilities and lack of formal autopsies.

**Table 3:** Fetal/infant deaths during gestation and the first 12 months of life (N = 75).

Timing of death	n	%
Miscarriage (<22 weeks’ gestation)	7	9.3
Stillbirth (≥22 weeks gestation to term)	33	44.0
Neonatal (birth to 28 days)	17	22.7
Post-neonatal (>28 days to 12 months)	18	24.0

**Fig 3:**
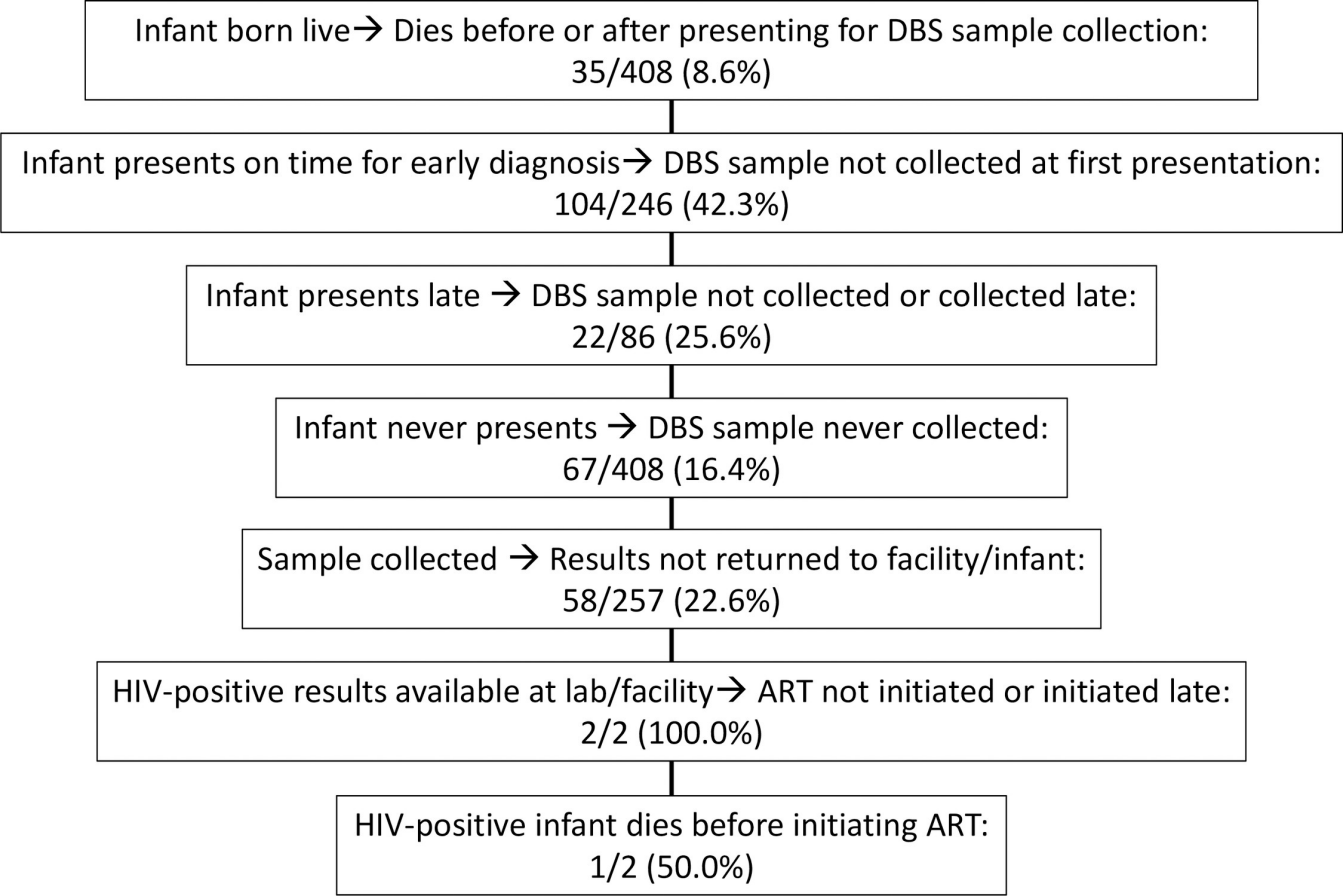
Major missed opportunities in MoMent’s early infant diagnosis cascade.

A summary of missed EID opportunities which occurred among the MoMent HEI cohort are presented in Fig 3.

## Discussion

This paper describes early HIV diagnosis and treatment linkage outcomes for HIV-exposed infants of pregnant women enrolled in the MoMent Nigeria study. It presents a granular assessment of programmatic missed opportunities that go beyond timely EID presentation. Even though our findings suggest that the structured Mentor Mother intervention improved infant presentation for testing and ultimately sample collection, this behavioral intervention cannot comprehensively address all missed EID opportunities.

Only a few prior studies have described gaps and missed opportunities in Nigeria’s PMTCT program. Adeyinka and colleagues performed a secondary analysis of data collected from Nigeria’s national PMTCT program between 2008 and 2014 [36]. Their findings highlighted gaps in ARV coverage for pregnant Nigerian women identified as HIV-positive [36]. Their analysis was however limited in that it included only maternal data and did not provide any data on HEI outcomes. Our analysis from the MoMent study, albeit limited to rural communities in two states, provides details on outcomes among >400 HEI from birth up to 12 months of life.

The Baby Shower study in Enugu state, South-Eastern Nigeria reported on outcomes for 69 live-born HEI [37]. Forty-three (62.3%) of these HEI received EID within two months of age; however it took an average of 5.6 months for results to be received. Aliyu et al reported EID enrollment as a function of DBS sample collection for infants of 712 HIV-infected women in North-Central Nigeria [38]. Only 357 women (50.1%) in their study cohort ever had DBS samples collected for their infants [38]. Earlier maternal enrollment in PMTCT care, enrollment at urban vs rural clinics and maternal receipt of combination ART were reported as predictors of EID enrollment [38].

Studies from other sub-Saharan African countries have highlighted similar missed EID opportunities, albeit with somewhat better coverage/uptake. Woldesenbet et al reported that only 62% of 2,856 South African HEI had mothers intending to request for EID or had the required EID service documentation [39]. From Burkina Faso, Coulibaly et al reported that only 29.4% of 1,064 HEI received a virologic test within 18 months of life, with 97.8% receiving results by four months post-sample collection [40]. In Ethiopia, 66.2% of 435 HEI had DBS samples collected by 8 weeks of life, and 3.5% had no evidence of sample collection after 12 months of follow-up [41]. Approximately 81% of these HEI received DNA PCR results within 3 months, with 16.1% never receiving results. All of the aforementioned studies have analyzed for missed EID opportunities, however they focused on receipt of EID testing. Presentation without sample collection-as we have outlined- has not been previously examined in detail.

We have previously reported formative study data on barriers to efficient EID service delivery in our Nigerian study setting, which include poor provider attitude, stock-out of DBS commodities, collection of DBS samples only on few designated days, and human resource constraints [42]. Similar EID barriers have been reported from other African countries; additionally, client time/cost of travel and services at facilities, poor EID data documentation/management and weak linkage of PMTCT/EID with ART and maternal-child health services have been identified [43–45].

A quarter of infants in our study presented after two months of age. This is lower than the reported “late EID uptake” of ~30–40% from missed opportunity studies from Ethiopia [41] and South Africa [39]. However, these studies did not ascertain how many of these “late EID uptake” cohorts were “failures to present early” versus “failures to test at first presentation.” For HIV-infected infants, delayed diagnosis is a missed opportunity for initiating early treatment and preventing excess mortality. We posit that health facilities’ failure to provide same-day DNA PCR testing for appropriately-presenting HEI constitutes a neonatal third delay, resonant with Thadeus and Maine’s “Three Delays” maternal mortality framework [46].

Besides the missed opportunities of “failures to present early” and “failures to test at first presentation”, there is the missed opportunity of “not presenting at all.” In our study, 67 out of 408 HEI (16.4%) never presented for DBS sample collection; this included 21 infants who died within the 12-month follow-up period. The Baby Shower trial cited 16% (11/69) mortality and 13% (9/69) loss to follow-up among HEI followed-up for 18 months in South-Eastern Nigeria [37]. While the reasons for non-presentation and causes of death for MoMent and the aforementioned studies were not clear, these events have to be considered missed opportunities for successful EID and ART initiation for HEI who may have been HIV-infected. Detailed patient-, facility-, health system- and community-level root-cause analyses are needed to elucidate why these infants never presented, or died before presentation.

### Impact of study findings on the Nigeria PMTCT program

The results of this study suggest structural failures in Nigeria’s HIV response, especially as it relates to PMTCT. In addition to previously-known maternal missed opportunities, MoMent demonstrates that the infant cascade has not fared much better, with nearly 1 in 4 DNA PCR results in our cohort not being received up to more than two years post-collection, even when samples were collected within two months of birth.

There is a clear disconnect between what should be national policy and what prevails at the clinical interface level. Whereas interventions such as mentor mothers can improve uptake of PMTCT and bring mothers and infants back to facilities for EID, they cannot address the missed opportunities of the 1 in 4 babies in this study that never had a DBS sample collected despite presentation, or the nearly 1 in 6 babies that would have had to return more than once for sampling.

Although the two HIV-infected babies that died/were lost to follow-up had tests done, the lack of effective retention-in-care and follow-up programs is only too obvious and concerning. The national HIV program needs to review the impact of current policies and guidelines for PMTCT and EID, taking cognizance of this study’s findings to address preventable leakages along the PMTCT/EID cascade.

### Study limitations

Our study has some limitations. We were unable to characterize in numerical detail the loss of samples and delays in the return of results within the study follow-up period. Estimation of turn-around time for result delivery to the patient includes dates of result receipt at the healthcare facility. We are limited in that we lacked comprehensive data for all study HEI to analyze turn-around-time; part of the issue was poor data documentation between labs and healthcare facilities in terms of reporting and receipt (or lack thereof) of DNA PCR results. This in itself is a missed opportunity and may have led to underestimation of infant HIV positivity rate. We did not evaluate missed opportunities due to sample rejection because of poor documentation, nevertheless, DBS sample rejection rates in our study setting have been documented [47]. Additionally, we were unable to collect final outcome data on HIV status for HEI at 18 months of age due to limitations in the study timeline which restricted participant follow-up to only 12 months post-delivery. Lastly, we did not have detailed mortality data to report attributable causes of death for presenting and non-presenting infants who died. It would be important to investigate and determine attributable causes of mortality among HEI and to describe contributing factors.

## Conclusions

Given Nigeria’s importance in the African regional and global agenda for the prevention and elimination of MTCT, persistently slow progress in this country is of particular concern. Point-of-care DNA PCR testing and SMS (short message service) printers could improve access to EID, timely receipt of results and infant ART initiation [1, 48–50]. However, these innovations may not be useful if testing commodity availability/human resources for DBS sample collection, and access to infant ART are not concurrently optimized through quality improvement approaches. Ultimately, structural interventions should be implemented to complement impactful behavioral interventions, and any missed opportunity for EID and initiation of infant ART should be considered a sentinel event.
